# Proteomic analysis of plasma-derived extracellular vesicles: pre- and postprandial comparisons

**DOI:** 10.1038/s41598-024-74228-4

**Published:** 2024-10-03

**Authors:** Alejandra P. Garza, Elisa Wider-Eberspächer, Lorena Morton, Marco van Ham, Éva Pállinger, Edit I. Buzás, Lothar Jänsch, Ildiko R. Dunay

**Affiliations:** 1 Institute of Inflammation and Neurodegeneration, Medical Faculty, Otto-Von-Guericke University, Magdeburg, Germany; 2 Cellular Proteome Research Group, Helmholtz Centre for Infection Research, Braunschweig, Germany; 3 Department of Genetics, Cell- and Immunobiology, Semmelweis University, Budapest, Hungary; 4 HCEMM SU Extracellular Vesicle Research Group, Budapest, Hungary; 5 HUN-REN-SU Translational Extracellular Vesicle Research Group, Budapest, Hungary; 6 Center for Behavioral Brain Sciences, Magdeburg, Germany; 7 Center for Intervention and Research on Adaptive and Maladaptive Brain Circuits Underlying Mental Health (C-I-R-C), Jena-Magdeburg-Halle, Germany

**Keywords:** Postprandial, Extracellular vesicles, Plasma, Liquid biopsy, Proteomics, Fasting, Proteomics, Biomarkers

## Abstract

Extracellular vesicles (EVs) are key in intercellular communication, carrying biomolecules like nucleic acids, lipids, and proteins. This study investigated postprandial characteristics and proteomic profiles of blood-derived EVs in healthy individuals. Twelve participants fasted overnight before baseline assessments. After consuming a controlled isocaloric meal, EVs were isolated for proteomic and flow cytometric analysis. Plasma triacylglyceride levels confirmed fasting completion, while protein concentrations in plasma and EVs were monitored for postprandial stability. Proteomic analysis identified upregulated proteins related to transport mechanisms and epithelial/endothelial functions postprandially, indicating potential roles in physiological responses to nutritional intake. Enrichment analyses revealed vesicle-related pathways and immune system processes. Flow cytometry showed increased expression of CD324 on CD9^+^CD63^+^CD81^+^ large extracellular vesicles postprandially, suggesting an epithelial origin. These findings offer valuable insights into postprandial EV dynamics and their potential physiological significance, highlighting the need for stringent fasting guidelines in EV studies to account for postprandial effects on EV composition and function.

## Introduction

Extracellular vesicles (EVs) have garnered significant attention in the scientific community due to their critical roles in intercellular communication and their potential use as diagnostic and therapeutic agents^1–4^. These robust particles, released by various cell types, carry a diverse and mixed cargo of proteins, nucleic acids, and lipids, reflecting the physiological state of their parent cells^5–7^. EVs are found in various bodily fluids, including urine, saliva, amniotic fluid, cerebrospinal fluid, serum, and plasma^8–13^. Among these, blood and its derivatives are the most frequently utilized biofluids in research due to their ease of access through minimally invasive and well-established procedures^14–16^.

EVs are commonly categorized by their size, which is one of their key characteristics. According to the most recent position paper from the International Society of Extracellular Vesicles^17^ "large extracellular vesicles" (lEVs) are defined as EVs larger than 200 nm, whereas “small EVs” are those smaller than 200 nm. Therefore, in this study, for flow cytometry analysis, we refer to our EV population as “lEVs” to denote those within the 300–1000 nm range. For analyses conducted using other methods, we use the term “EVs” to encompass the broader range of vesicle sizes obtained.

The study of EV dynamic compositions in the context of physiological stimuli, such as fasting and postprandial states, is of considerable interest due to their physiological functions such as platelet biology, inflammation and immunity, the regulation and maintenance of blood pressure, regulation of water and salt levels in urine, pregnancy, fertilization and implantation among others, and potential clinical applications, particularly concerning liquid biopsies and cargo and delivering of therapeutic molecules^4,18^. Due to the possible influence of fasting on the isolation, composition and abundance of EVs, a growing interest in the characterization of plasma-derived EVs pre- and postprandially has been developed. For instance, previous studies have shown that an isocaloric low-fat meal did not increase blood-derived endothelial microparticles (EMPs)^19^, whereas a high-fat meal did. Recent literature indicates that, while the total quantity of plasma-derived EVs may not show noticeable differences between fasted and postprandial states, important changes have been observed in lipid content and EV-size analyzed by nanoparticle tracking analysis (NTA), acknowledging the challeges entailed with EV separation from peripheral blood that are attributed to the presence of lipoproteins of similar size^20–22^. Additionally, another study found no differences in small non-codign RNAs extracted from EVs between fasting and postprandial samples, suggesting that fasting status would not be a relevant variable when particulary studyin sncRNAs from EVs^23^. Given the importance and widespread use of proteome analysis in EV research, we shought to determine weather fasting status affects this particular aspect since, to the best of our knowledge, no previous studies have addressed this question.

Various techniques for separating EVs offer unique strengths and limitations that vary based on the specific research objectives^24^. Recently, we have demonstrated the efficacy of using differential centrifugation to enrich EVs in plasma samples, particularly when preparing samples for flow cytometry analyses^25^. To study whether fasting is necessary before sample collection for a proteomic analysis, we aimed to investigate the impact of fasting status on (i) EV abundances and (ii) protein content and composition. This investigation is particularly pertinent given the challenges associated with implementing fasting in specific research contexts, potentially posing constraints within heterogeneous population samples, such as pediatric studies, and in individuals with metabolic conditions. Despite the recognized need, standardized guidelines regarding fasting status for the accurate characterization of plasma-derived EV proteome are yet to be established.

## Results

### No alteration in plasma and EV protein concentrations during the post-prandial state

**Fig. 1:**
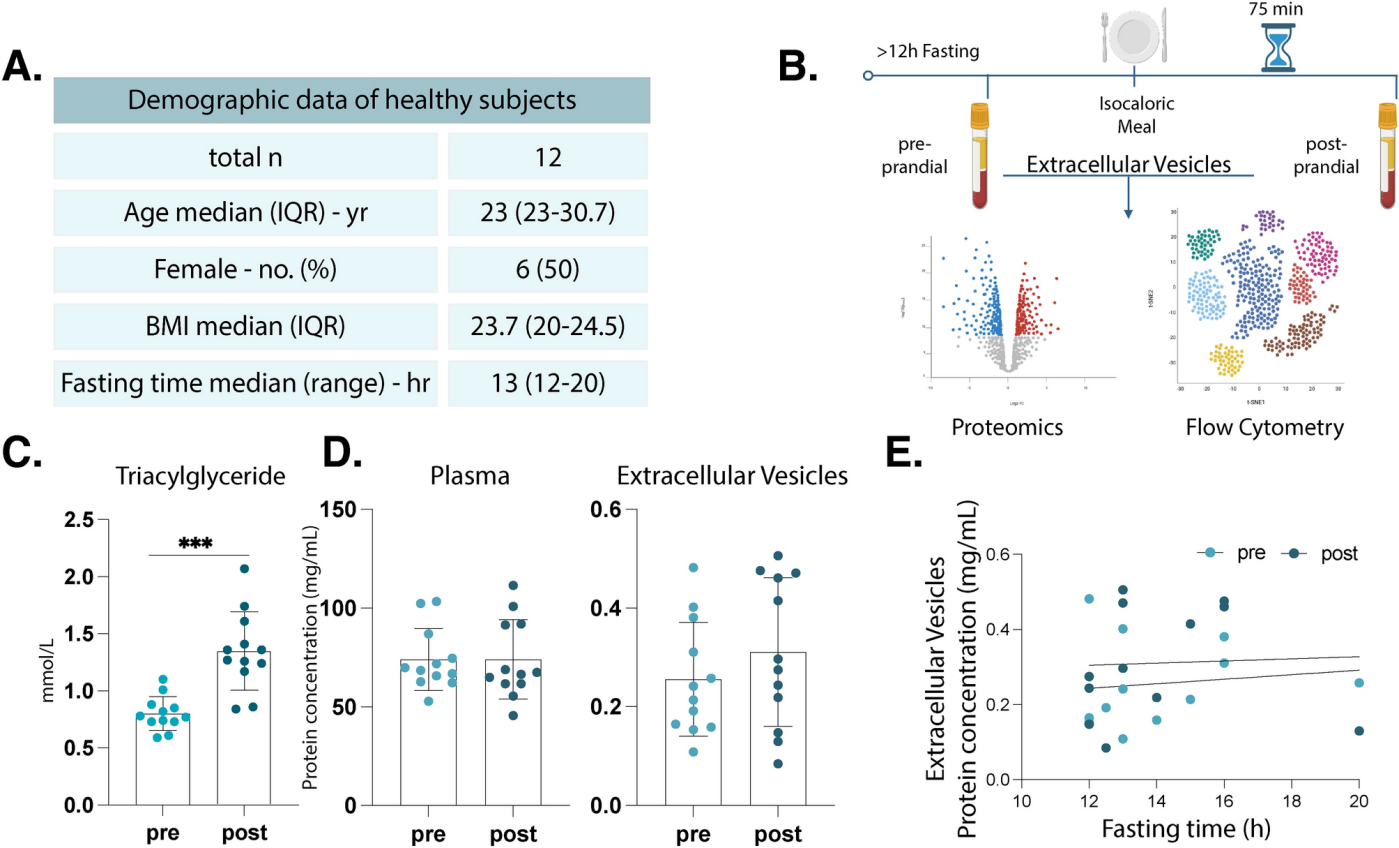
Experimental design and pre-requisites for assessing postprandial changes in healthy individuals. Detailed demographic information of the healthy participants (**A**). Schematic representation of the experimental setup, wherein blood samples were collected from healthy participants before and 75 min after consumption of an isocaloric meal. Samples were then processed to obtain plasma and plasma EVs, of which the later were used for detailed characterization by proteomics and flow cytometry (**B**). Bar chart illustrating the triacylglycerides levels in plasma before and after food intake, indicating postprandial changes (**C**). Bar plots displaying total protein concentrations in plasma and plasma-EVs, both pre- and postprandially (**D**). Correlation plot demonstrating no correlation between fasting time and total protein concentration in EV samples both in pre- and postprandial time points (**E**). n = 12, all samples were measured in technical triplicates. Statistical analyses were performed using paired t-test (**C**–**D**) and Pearson correlation (**E**), respectively. *P* values: * for *p* ≤ 0.05; ** for *p* ≤ 0.001; *** for *p* ≤ 0.0001.

We examined the postprandial alterations in plasma-derived EVs within a cohort of twelve healthy participants (median age 25 ± 3.6 years old, 6 females, median body mass index (BMI) 23.78 ± 2.7). Participants were instructed to undergo an overnight fasting period (median 13 ± 2.3 h) (Fig. 1A), where plasma and plasma-derived EVs were isolated for baseline assessments (referred to as the “pre” group). Subsequently, an isocaloric meal was administered, and after a 75-min interval, a second round of blood sample collection (referred to as the “post” group) was conducted. After all samples were collected, EVs were applied for a comprehensive analysis utilizing proteomics and multicolor flow cytometry. This experimental setup (Fig. 1B) aimed to discern the impact of a standardized meal on the compositional and quantitative aspects of EVs in the pre-and postprandial state, contributing to our understanding of the EV dynamic response to nutritional stimuli. Triacylglycerides (TAGs) are known to increase in the post-prandial state following an overnight fast, which we could validate with a significant increase in plasma TAG levels measured at 75 min postprandially (pre, 0.8008 ± 0.1472 mmol/L; post, 1.349 ± 0.3439 mmol/L, *p* =  < 0.0001) (Fig. 1C).

Subsequently, we assessed the protein concentrations in plasma and plasma-derived EVs at both pre-and postprandial time points. The outcomes indicated that protein levels remained relatively stable between both conditions in plasma (pre, 73.96 ± 15.7 mg/mL; post, 73.96 ± 20.04 mg/mL, *p* = 0.9097) and EVs (pre, 0.2558 ± 0.1155 mg/mL; post, 0.3106 ± 0.1505 mg/mL, *p* = 0.3275), as depicted in Fig. 1D. Given the variability in fasting durations among participants, we conducted a correlation analysis to investigate potential associations between fasting times and changes in protein levels. Our analysis revealed no significant correlation between fasting duration and the protein concentrations in plasma-derived EVs in both pre- (*r* = 0.1241, CI = − 0.4843 to 0.6516, *p* = 0.7008) and postprandial (r = 0.0452, CI = − 0.5427 to 0.6035, *p* = 0.8889) conditions (Fig. 1E).

## Proteomic insights into postprandial extracellular vesicle dynamics

**Fig. 2:**
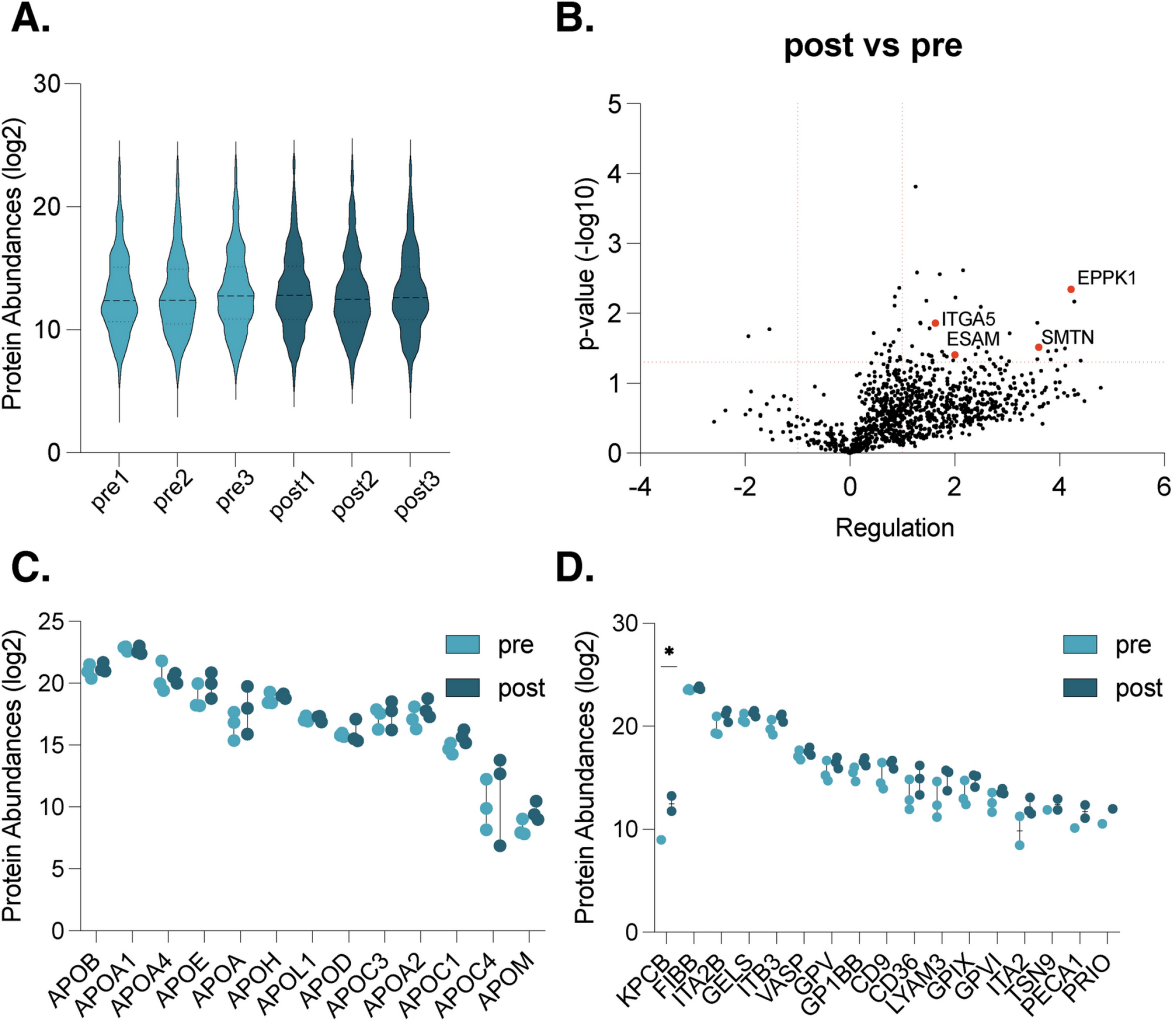
Proteomic analyses of fasted and postprandial isolated extracellular vesicles. Distribution of relative protein abundances (log2) in isolated EV samples (**A**). Volcano plots showing protein regulations (− log10) in EVs isolated post- vs preprandially (**B**). Protein abundances of apolipoproteins (log2) in EV samples comparing pre- and postprandial conditions (**C**). Platelet protein abundances (log2) in EV samples comparing pre- and postprandial conditions (**D**). n = 3, all samples were measured in technical triplicates; *P* values are given in − log10. Student’s t-test using an FDR of 0.05 (= 1.3 − log10) and a up- or down regulation of < − 2 or > 2 (≤ − 1 or > 1 log2).

To gain a comprehensive understanding of the protein composition within our EV samples, three participants were randomly selected to conduct an in-depth assessment of EV protein content utilizing proteomic techniques and mass spectrometry in pre- and post prandial conditions. This approach facilitated a detailed exploration into the protein distribution of 2230 proteins across samples, revealing consistent protein abundances in all samples (Fig. 2A and Supplementary Table 1). Subsequently, we investigated differentially regulated proteins between pre- and postprandial states in healthy participants. For that, and to enhance robustness of our data, only proteins that were identified and quantified in at least two samples of either the pre- or postprandial group were considered as valid. Differentially expressed proteins were identified by performing the Student’s *t*-test and using an FDR of 0.05 (= 1.3 − log10) and an up- or down regulation of < − 2 or > 2 (= < − 1 or > 1 log2) . Generally, we found more proteins to be upregulated in EVs at the postprandial time point (Fig. 2B). Notably, several proteins implicated in epithelial and endothelial cell functions exhibited significant upregulation in the postprandial state, including EPPK1 (epiplakin), ITGA5 (integrin sub-unit alpha 5), SMTN (smoothelin), and ESAM (endothelial cell adhesion molecule) (Fig. 2B), emphasizing their potential role in postprandial physiological processes. Due to the difficulties separating lipoproteins from EVs, we evaluated the main apolipoproteins associated with food intake (Fig. 2C). We assessed the abundance of ApoB, ApoA1, ApoA4, ApoE, ApoA, ApoH, ApoOL1, ApoD, ApoC3, ApoA2, ApoC1, ApoC4 and ApoM and found similar levels between pre- and post conditions (Fig. 2C). Additionally, we explored whether the presence of platelet proteins varied depending on fasting status. We analysed 24 reference platelet proteins^26^, out of which we found 17 present in our sample: KPCB (Protein kinase C beta), FIBB (Fibrinogen beta chain), ITA2B (Integrin subunit alpha 2b), GELS (Gelsolisin), ITB3 (Integrin subunit beta 3), VASP (Vasodilator stimulated phosphoprotein), GPV (Platelet glycoprotein V), GP1BB (Glycoprotein Ib platelet subunit beta), CD9, CD36, LYAM3 (P-selectin), GPIX (Glycoprotein IX platelet), GPVI (Glycoprotein VI platelet), ITA2 (Integrin subunit alpha 2b), TSN9 (Tetraspanin 9), PECA1 (Platelet and endothelial cell adhesion molecule 2) and PRIO (Prion protein). While these proteins have been shown to be present in platelets, it is important to note that our samples are enriched for EV and not platelets, and these markers are predominantly not exclusive to platelets. Our analysis unvailed a significant upregulation of only one protein, protein kinase C beta type (PKCB), in the postprandial group (*p* = 0.0386) (Fig. 2D). Notably, this protein plays a role in various biological processes, including B-cell activation and the regulation of endothelial cells in the brain microvasculature and its expression occurs in cells other than platelets^27,28^.

### Enriched gene ontology (GO) and reactome pathways analysis

**Fig. 3:**
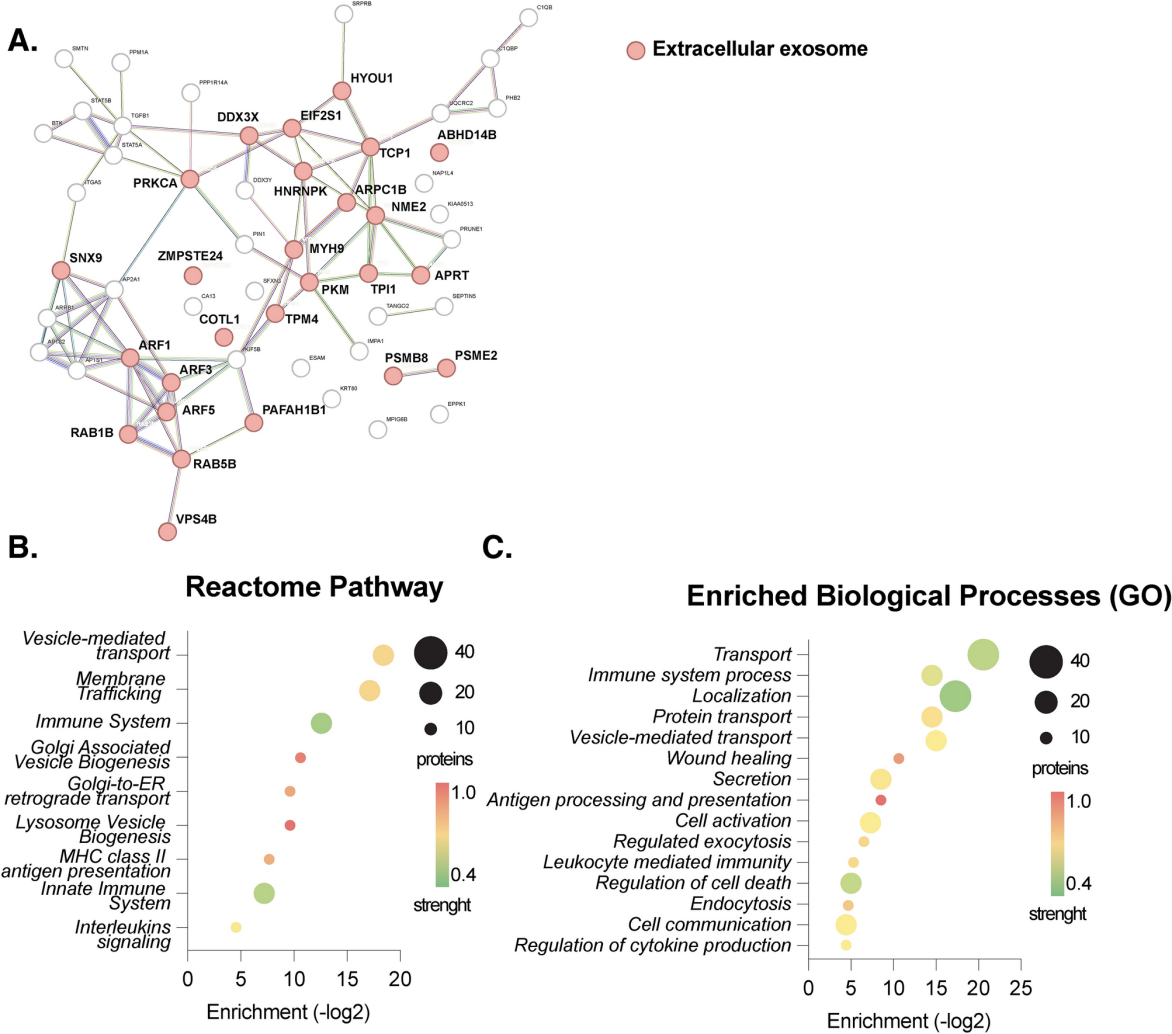
Enrichment analyses of significantly regulated proteins in the posprandial state. Network analysis showing proteins annotated as “extracellular exosome” in red, names are given in bold (**A**). Enrichment analyses showing enriched reactome pathways and biological processes (GO) analyses revealed multiple enriched biological processes (*p*-values given in − log2) in postprandial EVs. Size of the dots indicate number of proteins; color of the dots indicates strength of the enriched processes and pathways (**B**,**C**). Analyses were performed using the STRING database.

To identify prominent biological processes and pathways involved in EV dynamics, we conducted protein enrichment analyses using the differently abundant EV proteins when comparing the pre- and postprandial states (Fig. 3). First, utilizing differentially expressed proteins with regulation lower than − 2 and/or higher than 2 and *p* value above 1.3 an in-depth network analysis of the interaction between the differentially expressed proteins utilizing the STRING database was performed, revealing significant enrichment within the “Extracellular exosome” network in the postprandial state (Fig. 3A). Further analysis of enriched reactome pathways and biological processes (GO) in our sample unveiled an expected emphasis on vesicle-related pathways, encompassing transport and membrane trafficking in the postprandial samples such as ARF3 and ARF5 (ADP ribosylation factor 5), MYH9 (Myosin heavy chain 9), PSME2 (Proteosome activation subunit 2), TGFB1 (Transforming growth factor beta 1), SFXN3 (Sideroflexin 3), VPS4B (Vacuolar protein sorting 4 homolog B), EIF2S1 (Eukaryotic translation initiation factor 2 subunit alpha), COTL1 (coactosin like F-aciting binding protein 1), among others (Supplementary Table 1). Moreover, pathways and biological processes related to the immune system emerged prominently within the enriched pathways and processes (Fig. 3B,C). Detailed examination of the proteins associated with these processes revealed a subset of proteins intricately involved in both epithelial and endothelial functions (Fig. 2B).

### Increased EV surface expression of epithelial marker CD324 on postprandial lEVs

**Fig. 4:**
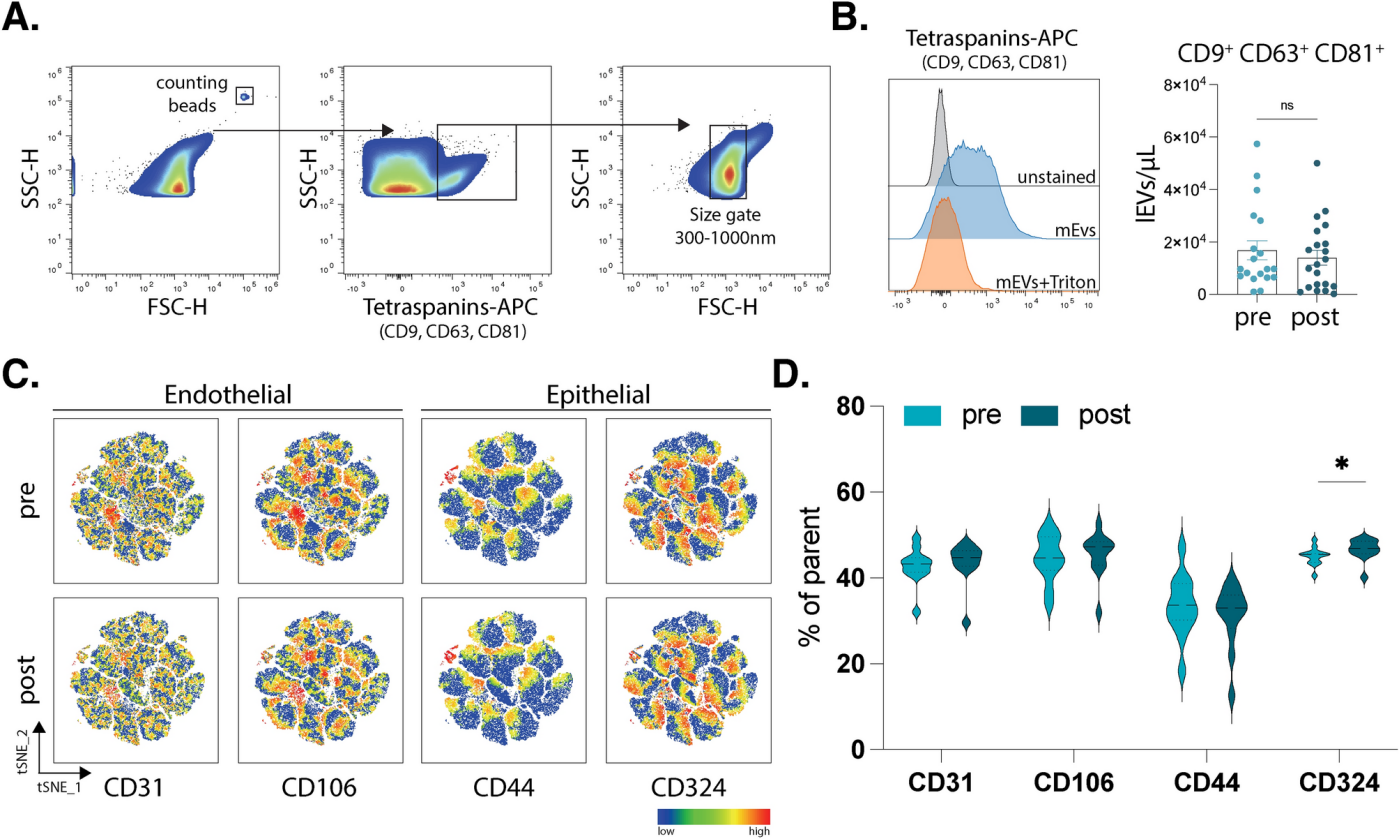
Flow cytometry of plasma extracellular vesicles. Representative gating strategy of plasma derived lEVs showing first counting beads as reference (left graph). Selection of events positive for tetraspanin markers (CD9, CD63 and CD81) was followed by selection of events based on a size gate established with silica beads, additionally showing sensitivity to Triton X and an unstained control (**A**). Bar charts showing absolute number of lEVs per microliter comparing pre- and postprandial timepoints based on measurement with counting beads (**B**). t-distributed stochastic neighbor embedding (t-SNE) plots showing the mean fluorescence intensity of CD31, CD106, CD44, and CD324 in a total of 80,400 positive events (based on CD9+ , CD63+ CD81+ events and size) in each pre- and postprandial states (**C**). Violin plots showing the frequency of positive events for CD31 and CD106 (endothelial markers) as well as CD44 and CD324 (epithelial markers) in pre- vs postprandial states (**D**). n = 12, all samples were measured in technical duplicates. Statistical analyses were performed using paired t-test. *P* values: * for *p* ≤ 0.05; ** for *p* ≤ 0.001; *** for *p* ≤ 0.0001.).

Given the crucial roles of intestinal and vascular barriers in facilitating inter-system communication, particularly during postprandial physiological processes, we hypothesized that the proteins identified in lEVs may play significant roles in this interaction. To investigate this further, we employed multicolor flow cytometry to analyze specific surface markers on lEVs associated with endothelial and epithelial cells (Fig. 4A,B). This approach aimed to elucidate the potential involvement of these vesicles in communication between the gut and peripheral systems following nutrient intake. We selected CD31 and CD106 as enriched expression markers on endothelial cells and CD44 and CD324 on epithelial cells. Our results showed no significant changes in the total concentration of EVs per microliter between the pre- and postprandial states (pre, 16,890 ± 3630 lEVs/μL; post, 14,003 ± 2834 lEVs/μL, *p* = 0.6878) showing a clear correlation with the protein levels assessed via colorimetric assay in which also no changes were present (Fig. 1D). A visualization of the relative expression of these markers in our EV sample was performed (Fig. 4C) showing a higher expression of CD324 and CD106. When quantifying the actual frequency of these markers on the lEVs surface, we observed a significant increase in the postprandial expression of CD324 on EVs (tetraspanin^+^, size gate^+^), a marker known for its role in cell–cell adhesion, mobility, and proliferation of epithelial cells (Fig. 4D) (pre, 45.03% ± 2.102; post, 46.64% ± 2.45; *p* = 0.0294). In order to assess the abundance of these markers on the EV’s surface, mean fluorescence intensity (MFI) was also assessed, and despite observable differences in the frequency of CD324, no significant dfferences were present when comparing MFI signals (Supplementary Fig. 2). In conclusion, our findings demonstrate a significant increase in postprandial surface expression of the epithelial marker CD324 on lEVs, supporting the hypothesis of their engagement in communication with epithelial cells following food intake.

## Discussion

In this study, plasma concentrations and the molecular composition of EVs were assessed in postprandial states of healthy individuals. Our results offers new perspectives into the proteomic landscape and changes in specific surface markers on EVs in response to nutritional intake with potential consequences for biomarker research.

Previous research assessing plasma-derived microparticles (MPs) or more recently, EVs has shown an increased concentration postprandially^29–31^. In our study, we evaluated potential differences using a colorimetric assay and found similar protein concentrations in plasma and EVs in both fasting and postprandial samples. Upon targeted flow cytometry evaluation using tetraspanin markers, no significant alterations were detected in the total number of lEVs. It is important to note that previous studies employed different isolation methods and used various markers in flow cytometry to indicate the presence of these particles. Additionally, we must consider the possibility of lipid co-measurement, as they share similar characteristics with EVs or MPs, and could have been detected as MPs in earlier studies^21^. Given the variations in isolation and assessment methods, the evolving nomenclature, and advancements in this rapidly growing field, our results should be interpreted within the context of plasma-EVs isolated using a streamlined centrifugation process and assessed with tetraspanin markers.

Addionally, our analysis revealed that EVs maintain a consistent protein concentration, regardless of the fasting duration, and indicate a tightly controlled homeostatic mechanism with no significant effect in response to short-term changes in nutritional status. From a physiological perspective, the stability of EV protein levels despite fasting states emphasizes the EVs potential to serve as stable carriers of biological signals, of particular importance in the context of diagnostic biomarker development, where EVs can provide a reliable snapshot of biological states independent of a fasting period. For clinicians and researchers, it suggests that EV-derived biomarkers may not require strict controls for fasting duration, simplifying the sample collection protocol and expanding the applicability of EV-based diagnostics. Still, is recommended that studies involving plasma-derived EVs report fasting status if known, along with the duration of the fasting period^16^. Our present results demonstrate that quantitatively via flow cytometry using specific EV identification markers, there are no significant fluctuations observed in the number of blood EVs concerning fasting status.

While our results revealed that the overall EV numbers in plasma remained relatively stable before and after the intake of an isocaloric meal, it is crucial to distinguish this overall stability from the changes observed at the proteomic level. Our proteomic data indicates the enrichment of an “Extracellular Exosome” network, affirming the effectiveness of our isolation method in yielding a sample enriched with EVs. Further, our proteomic analysis revealed a distinct landscape of EV protein composition, characterized by a significant postprandial upregulation of proteins associated with transport mechanisms, as well as proteins integral to epithelial and endothelial cell functions. This upregulation indicates that, although the total protein content may remain unchanged, the proportion of specific types of proteins within the EVs and the prevalence of certain EV subpopulations is altered in response to food intake. These findings suggest a selective enrichment or mobilization of EVs carrying proteins crucial for postprandial physiological processes, reflecting an adaptive response to such a metabolic state.

Recent studies have delved into the multifaceted roles of EVs across physiological and pathological processes, indicating their critical function in nucleic acid and protein transfer, receptor sequestration, interaction with the extracellular matrix (ECM), and their emerging role in endocrinology and tissue repair^32,33^. Other omics approaches, such as lipidomics, have also proven helpful in the understanding of EV function in homeostasis and metabolic changes^34^. Collectively, these studies elucidate the mechanism through which EVs promote intercellular signaling and are consistent with our findings of postprandial EV dynamics where we have observed a marked increase in proteins linked to transport and cellular communication. The identification of enriched gene ontology (GO) and Reactome pathways related to vesicle-mediated transport, membrane trafficking, and immune system processes further emphasizes the complex involvement of EVs in systemic physiological responses.

A notable challenge in isolating EVs from plasma is the presence of large lipoproteins, such as low-density lipoprotein (LDL), which share physical characteristics with EVs^21^. LDL predominantly comprises Apolipoprotein B (ApoB). Thus, we evaluated its presence, along with various other apolipoproteins, in our EV sample, and our findings revealed no significant changes between the two conditions, indicating a minimal impact of fasting on these particular lipoproteins in EV samples. Another potential concern in EV isolation is contamination with platelets. To address this, first we evaluate to methods of isolating PPP, assessed platelet presence via flow cytometry, and observed no significant differences in platelet quantity between the two methods (Supplementary Fig. 1). It is worth highlighting that various protocols exist for PPP isolation, with Method 2 (2x, 2500 g for 20 min) being the most widely accepted within the EV community^35,36^. Although our results did not show significant changes, a discernible trend was present suggesting the presence of platelets in our sample, which could potentially affect downstream analyses. Therefore, for consistency and reproducibility, we recommend adhering to the Method 2 and the general guidelines outlined by the International Society for extracelluar vesicles (ISEV)^24^.

The analysis of proteins implicated in enriched networks revealed heightened levels of endothelial and epithelial-related proteins. This finding holds particular significance given the pivotal roles of both intestinal and vascular barriers in mediating inter-system communication, particularly in the context of gut-periphery interactions during physiological processes^37^. Notably, the coordinated postprandial upregulation of epiplakin (EPPK1), a protein predominantly expressed in epithelial and glandular cells that are also present in the gastrointestinal tract, and integrin sub-unit alpha 5 (ITGA5), an integrin primarily expressed in intestinal tissue and endothelial cells, suggests a coordinated gastrointestinal response detectable in lEV composition^38,39^. Similarly, the postprandial upregulation of smoothelin (SMTN) and endothelial cell adhesion molecule (ESAM) postprandially underscores the potential involvement of EV-mediated communication between the digestive system and peripheral tissues^40–42^. These observations hint at an unexplored mechanism of communication facilitated by EVs following food intake involving endothelial and epithelial, affirming further investigation.

To investigate this, we evaluated the surface expression of epithelial and endothelial markers on lEVs via flow cytometry. Our results revealed an increased surface expression of the epithelial marker CD324 on lEVs postprandially. CD324 (CDH1, cadherin 1) is highly expressed in the intestinal epithelium, suggesting that the presence of CD324^+^ lEVs in peripheral circulation could indicate that epithelial cells in the gut release EVs in response to nutrient contact. This may play a role in regulating the intestinal barrier and nutrient metabolism. Additionally, it has been described that lEVs can carry a wide array of bioactive cargoes such as proteins, lipids, organelles, and amino acids^2,6,37,43^. Therefore, one could hypothesize that EV signaling is part of the physiological response to food intake and the subsequent metabolic changes, with potentially multisystemic targets such as the liver, pancreas and adipose tissue. These EVs might also be involved in local targeting, such as signaling for epithelial barrier opening, nutrient absorption, epithelium remodeling, or even modulation of motility patterns and luminal flow. Our findings align with the overall biological processes enriched in our postprandial samples, including transport, protein transport, membrane trafficking, and cell communication. Additionally, the capabilities of EV cargo in clinical diagnostics, as discussed by Qian et al.^43^, parallels our detection of specific postprandial alterations in EV protein composition, highlighting the diagnostic and therapeutic potential of understanding EV protein shift in response to food intake. Finally, our proteomic analysis of EVs reveals several postprandial changes related to transport and communication. Therefore, it is advisable to adhere to current guidelines that mandate reporting the fasting status of study participants and to consider the potential alterations that may arise depending on the downstream applications and focus of each study. Our findings demonstrate upregulation in biological processes related to immunity, cellular transport, communication, wound healing, and interleukin signaling. These factors must be considered in relation to the particular aspects under investigation.

The growing body of evidence regarding EVs and their role in intercellular communication further corroborates the importance of our findings, suggesting that postprandial EV profiles may be crucial in systemic metabolic regulation and intercellular communication^44,45^. Collectively, our results further unravel EV behavior and their physiological adaptation in postprandial states. By directly addressing previously identified gaps in the literature regarding the influence of fasting on EV phenotypes^16^, our results improve EV research methodologies and their physiological relevance at a proteomic level.

Our study employs a comprehensive and straightforward approach to investigate the effects of fasting on plasma concentrations and molecular composition of EVs encompassing analytical techniques such as flow cytometry and proteomics. We addressed the current gap in the literature about the stability of plasma and EV protein concentrations in response to nutritional intake and shed light on the changes in EVs during fasting and postprandial states and informing future research guidelines. We further describe which EV-derived markers and proteins are differentially expressed in two conditions during homeostasis, thereby possibly simplifying sample collection protocols and expanding the applicability of EV-based diagnostics. Our study’s primary limitation is the relatively small sample size, which may influence the generalizability of our findings. While our analyses have provided estimates of statistical significance, the limited sample size may constrain the statistical power and affect the robustness of the results. Despite these constraints, our study offers valuable insights and findings. To strengthen the conclusions and enhance the reliability of the results, future research with larger cohorts is recommended. Such studies would provide greater statistical power and help validate and extend our findings. While this study employs established EV isolation protocols and identification techniques, such as differential centrifugation, flow cytometry, and mass spectrometry, each method has inherent limitations that may impact the accuracy and reproducibility of the results. Other available methods could enhance the robustness of our findings. For example, size-exclusion chromatography (SEC) could improve the number and specificity of isolated vesicles, however, the use of this method has also been linked to a higher lipoprotein contamination than differential ultracentrifugation. Additionally, nanoparticle tracking analysis (NTA) could offer more precise measurements of particle size. We aimed to utilize a streamlined approach to sample acquisition and analysis, prioritizing methods that facilitate broader applicability in EV research, while ensuring the reliability and relevance of our findings. Additionally, working with freshly isolated samples would be ideal to more accurately represent the in vivo physiological state, leading to more reliable and reproducible results. Overall, while this study provides valuable insights into the effects of fasting on EV proteomics, its findings should be interpreted cautiously considering the aforementioned limitations and within the context of our isolation and characterization methods.

## Methods

### Experimental setup and blood withdrawal

Twelve young healthy participants (6 females, 6 males, mean age ± SEM: 26.42 ± 1.05 years, range: 23–32 years) were prospectively recruited at the Medical Faculty of the Otto von Guericke University in Magdeburg, Germany. Inclusion criteria were age over 18 years, overnight fast of ≥ 12 h (no alcohol, sugar-containing beverages, and food consumption). Exclusion criteria were acute diseases, chronic conditions (cardiovascular, neurological, psychiatric), uncontrolled metabolic disease, pregnancy, current or recent smoking and alcohol abuse. Every participant gave written informed consent. To determine the effects of fasting, peripheral blood was collected in the fasting state (pre-group) to establish an individual baseline, after which a standardized meal was provided. The meal was approximately 915 kcal, consisting of 34.8% fat, 55.42% carbohydrates, and 9.78% protein. The second studied time point was 75 min postprandial (post). Blood was collected using a 21G winged butterfly needle (BD Vacutainer, Safety-Lock™) into two sterile acid citrate dextrose (ACD) BD Vacutainer tubes containing 1 ml of ACD, and one lithium heparin coated (LH PST™ II) BD Vacutainer from a cubital vein at both time points. Tubes were inverted twice to ensure the incorporation of the agents and whole blood. All samples were processed within 1 h of collection.

### Triacylglycerides measurement

Blood collected in lithium heparin-coated tubes was centrifuged within one hour at 1.5 × 10^3^ g for 10 min at 4 °C. Plasma was transferred to sterile tubes and stored at − 80 °C until further processing. Samples were sent to and measured by the Institute of Clinical Chemistry at the Medical Faculty of Otto von Guericke University.

### Extracellular vesicle isolation

First, two methods were investigated in order to obtain platelet-poor plasma (PPP). Method 1 consisted of two centrifugation steps of the ACD blood at 1,5 × 10^3^ g for 10 min at room temperature (RT). Method 2 consisted of two steps of ACD blood centrifugation at 2,5 × 10^3^ g for 20 min at RT. Further, 2 mL of PPP from Method 1 was then transferred to fresh clean tubes and centrifuged at 14 × 10^3^ g for 70 min at 4 °C. Further, the supernatant was carefully removed, and the pellet was resuspended in 200µL 0.22 µm filtered phosphate-buffered saline (PBS) without Ca2 + and Mg2 + (fPBS-/-) and vortexed to ensure the solution of the previously formed pellet. This step was followed by another round of centrifugation 14 × 10^3^ g for 70 min at 4 °C, after which the supernatant was removed, and the pellet was reconstituted in fPBS^-/-^. Samples were stored at − 80 °C until further experiments. With this method, we isolated extracellular vesicles (EVs) from a broad size range. However, due to flow cytometer limitations, we can reliably analyze only particles between 300–1000 nm. According to the most recent position paper from the International Society of Extracellular Vesicles^24^, "large extracellular vesicles" (lEVs) are defined as EVs larger than 200 nm, whereas “small EVs” are those smaller than 200 nm. Therefore, in our flow cytometry analysis, we refer to our EV population as “lEVs” to denote those within the 300–1000 nm range. For analyses conducted using other methods, we use the term “EVs” to encompass the entire range of vesicle sizes obtained.

### Protein concentration measurement

Heparin plasma and EVs were thawed on ice and vortexed before further processing. Bio-Rad protein assay^46^ was performed according to instructions provided by the manufacturer (500–0006 Bio-Rad). Briefly, EVs were diluted 1:100 and plasma was diluted 1:4000 both in distilled water. Bovine serum albumin (BSA, BioRad Laboratories, USA) 1 mg/ml was used to determine the standard curve. Bradford reagent was mixed 1:5 with the respective sample and added to a 96-well plate. Samples were measured at 595 nm using a SpectraMax M5e microplate reader (Molecular Devices LLC). Each sample and standard were measured as technical triplicates and averages were calculated for further analysis.

### Flow cytometry

Aliquots of EVs were thawed on ice and vortexed to ensure a homogenous sample. 50 µl per sample were added to a 96-well plate with 35 µl of fPBS^-/-^ per well. Diluted samples were stained with labelled tetraspanin marker-specific antibodies CD9, CD63, CD81 (Allophycocyanin, BioLegend, concentrations: 6 µg/mL, 200 µg/mL and 200 µg/mL, respectively), hyaluronic acid receptor CD44 (Alexa Fluor 700, BioLegend, concentration 300 µg/mL), CD324 (Peridinin chlorophyll protein-Cyanine 5.5, BioLegend, concentration 0.2 mg/mL) and endothelial markers CD106 (Phycoerythrin, BioLegend, concentration 25 µg/mL), CD31 (Fluorescein isothiocyanate, BioLegend, concentration 200 µg/mL) and incubated for 30 min at 4 °C. Following this, samples were centrifuged at maximum speed for 10 min, supernatant removed by careful pippeting and resuspended in 200 µL fPBS-/-. To confirm the presence and purity of EVs samples were prepared as described above and lysed for 10 min with 0.1% Triton X solution. To identify the events on the size range of EVs, 300 nm to 1000 nm size reference silica beads (CD Bioparticles) were used. For quantification of events, AccuCheck counting beads (Invitrogen) were added to each sample and calculations were performed following the manufacturer’s instructions following the formula: (number of AccuCheck counting beads per µl) * (number of total events counted—TritonX resistant events) / (total number of beads counted) = absolute count of events / µl (Fig. 4A,B). Samples were acquired using the AttuneNxT flow cytometer (ThermoFisher) equipped with a small particle side scatter filter. Sample acquisition speed was set to 25 µl per min, SSC-threshold was set to 0.18 × 10^3^ and FSC-threshold was set to 0.15 × 10^3^. Each sample measurement was followed by 10% bleach and filtered distilled water to prevent carryover from the previous sample. Data were transferred to FlowJo 10.9.0 and gated on tetraspanin positivity and FSC and SSC utilizing the previously established size gate (Fig. 4A). Further, plots were generated using t-distributed stochastic neighbor embedding (t-SNE) algorithm for which events were down sampled using the DownSample plug-in. 6,700 size and tetraspanin positive events per sample were used and concatenated followed by tSNE utilizing all compensated parameters for 1,000 iterations and perplexity of 20, approximate random projection forest (ANNOY) as KNN (k-nearest neighbor) and FFT (Fast Fourier Transformation) interpolation as gradient algorithm. Further statistical analyses were performed using GraphPad Prism 10.1.1. Data was assessed for normality of distribution using D’Agostino & Pearson and Shapiro–Wilk tests, and paired t-tests or Wilcoxon tests were performed accordingly.

### Proteomics

EVs were suspended in 200 µl PBS and stored for further proteomic analyses. For protein recovery, protein digestion and peptide clean-up, a slightly adapted SP3 protocol was applied^47^. For that, extracellular vesicle samples were diluted in lysis buffer to final concentrations of 2% sodium dodecyl-sulfate (SDS), 100 mM NaCl and 250 mM triethylammonium bicarbonate (TEAB), and were supplemented with complete protease inhibitor cocktail (Roche). Lysis was increased by sonication using 10 cycles of a standard 30 s on/off Bioruptor protocol (Diagenode). Proteins were reduced for 1 h at 55 °C using a final concentration of 5 mM Tris(2-carboxyethyl)phosphine P(CH_2_CH_2_COOH)_3_ (TCEP) followed by reduction for 30 min at room temperature using a final concentration of 10 mM s-methyl methanethiosulfonate (MMTS). Carboxylate beads (20 µl) were added, and proteins were allowed to bind overnight upon acetonitrile (ACN) addition to a final concentration between 50 and 60%. Beads were washed twice with 80% ethanol and once with 100% ACN and allowed to dry on air. Beads were suspended in 50 µl 200 mM TEAB containing 5 mM TCEP and 10 mM MMTS. Trypsin was added in a ratio of 1 µg trypsin to 30 µg protein and proteins were digested overnight at 37 °C while shaking. Peptides were allowed to bind overnight upon ACN addition to a final concentration of at least 90%. Beads were spun down and supernatants transferred to LoBind Eppendorf tubes and stored for further analyses. Beads were washed three times with 100% ACN. Peptides were eluted by using first 20 µl 2% Dimethyl sulfoxide (DMSO) followed by a second step with 20 µl Millipore H_2_O. Purified peptides were vacuum dried, and pellets were suspended in 40 µl 0.1% formic acid. For liquid chromatography–mass spectrometry (LC–MS/MS) analyses, 100 ng total peptide mixture of each sample was measured as triplicates on a Evosep One HPLC (Evosep) connected to a TimsTOFPro mass spectrometer (Bruker) equiped with PaSER Version 2022c (Bruker) for real-time database searches. Samples were applied onto C18 Evotips (EV-2001; Evosep) according to manufacturer’s protocol. The Evosep One HPLC was operated with the standard 60 sample per day method (21 min gradient at a flow rate of 1.0 µl/min; buffer A: 0.1% formic acid, buffer B: 0.1% formic acid in acetonitrile). For the TimsTOFPro mass spectrometer, the standard MSMS Bruker method “DDA PASEF method for short gradients with 0.5 s cycletime” was employed. MS settings were: scan begin 100 m/z; scan end 1700 m/z; ion polarity: positive; scan mode: PASEF. Tims settings were: mode custom; number of PASEF ramps: 4; charge minimum: 0; charge maximum: 5; 1/K0 start 0.75 V*s/cm2; 1/K0 end 1.4 V*s/cm2; ramp time: 100.0 ms; MS average: 1. For protein and peptide identification and quantification, raw data files were loaded and run on the PEAKS software (PEAKS studio Xpro, Bioinformatics Solutions) using following settings; parent mass error tolerance: 20.0 ppm; fragment mass error tolerance: 0.03 Da; enzyme: trypsin; max missed cleavages: 2; peptide length range: 6–45; fixed modifications: beta-methylthiolation (C) + 45.99; variable modifications: oxidation (M) + 15.99; database: human release 15 November 2021. Downstream analyses, data presentation and statistical analyses were performed using Perseus V2.0.9.0, STRING database version 12.0 and GraphPad Prism 9.3.1. The mass spectrometry proteomics data have been deposited to the ProteomeXchange Consortium via the PRIDE partner repository with the dataset identifier PXD055597.

## Supplementary Information

Supplementary Information 1.

Supplementary Information 2.

Supplementary Information 3.

Supplementary Information 4.

## Data Availability

The datasets used and/or analysed during the current study are available from the corresponding author on reasonable request. Additionally, the mass spectometry data are publicly accesible on ProteomXchange.

## Abbreviations

ACD: Acid citrate dextrose
ACN: Acetonitrile
ANNOY: Approximate random projection forest
Apo: Apolipoprotein
BMI: Body mass index
BSA: Bovine serum albumin
DMSO: Dimethyl sulfoxide
ECM: Extracellular matrix
EMP: Endothelial microparticle
EPPK1: Epiplakin 1
ESAM: Endothelial cell adhesion molecule
EV: Extracellular vesicle
FFT: Fast fourier transformation
FIBB: Fibrinogen beta chain
fPBS: Filtered PBS
GELS: Gelsolisin
GO: Gene ontology
GP1BB: Glycoprotein Ib platelet subunit beta
GPIX: Glycoprotein IX platelet
GPV: Platelet glycoprotein V
ISEV: International Society of Extracellular Vesicles
ITA: Integrin subunit alpha
ITGA5: Integrin sub-unit alpha 5
KNN: K-nearest neighbor
KPCB: Protein kinace C beta
LC–MS/MS: Liquid chromatography–mass spectrometry
LDL: Low-density lipoprotein
lEV: Large extracellular vesicle
LH: Lithium heparin
LYAM3: P-selectin
MMTS: S-Methyl methanethiosulfonate
MP: Microparticles
NTA: Nanoparticle tracking analysis
PECA1: Platelet and endothelial cell adhesion molecule 2)
PBS: Phosphate-buffered saline
PKCB: Protein kinase C beta type
PPP: Platelet-poor plasma
PRIO: Prion protein
RT: Room temperature
SDS: Sodium dodecyl-sulfate
SEC: Size-exclusion chromatography
SEM: Standard error of the mean
SMTN: Smoothelin
t-SNE: T-distributed stochastic neighbor embedding
TAG: Triacylglycerides
TCEP: Tris(2-carboxyethyl)phosphine P(CH_2_CH_2_COOH)_3_
TEAB: Triethylammonium bicarbonate
VASP: Vasodilator stimulated phosphoprotein
