# Supplementary material for: Proteomic analysis of plasma-derived extracellular vesicles: pre- and postprandial comparisons
Source: Sci Rep. 2024 Oct 3;14:23032. doi: 10.1038/s41598-024-74228-4 (PMC11450010; doi:10.1038/s41598-024-74228-4)
Supplement: Supplementary file 1 — Supplementary Information 1. [file 41598_2024_74228_MOESM1_ESM.docx]

**Supplementary Figure 1.**

**Comparison of platelet presence in two isolation methods.** Method 1 involved two centrifugation steps of the ACD blood at 1,5 x 10^3^ *g* for 10 min at room temperature (RT), while method 2 entailed two centrifugation steps of ACD blood centrifugation at 2,5 x 10^3^ *g* for 20 min at RT. Both methods were followed by two centrifugation steps of 14 x 10^3^ *g* for 70 min at 4 °C. n=4, all samples were measured in technical duplicates. Statistical analyses were performed using paired t-test.

**Supplementary Table 1.**

**Proteomic analyses of plasma derived extracellular vesicles.** File containing multiple tabs with detailed information on proteomics data. The data used for specific figures are marked accordingly within each tab. Each tab corresponds to different aspects of the study, providing comprehensive data supporting the findings presented in the manuscript. The proteomic dataset generated and analyzed during the current study will be publicly available on ProteomeXchange.

**Supplementary Figure 2.**

**Mean Fluorescence Intensity (MFI) of surface markers on lEVs**. Bar chart depicting the MFI levels of four surface markers (CD31 and CD106 (endothelial markers), and CD44 and CD324 (epithelial markers) on lEVs. Two groups are compared: preprandial (light blue) and postprandial (dark blue). Data represent the average MFI values for each markers in the corresponding group. Error bars indicate the standard error of the mean (SEM). n=12, all samples were measured in technical duplicates. Statistical analyses were performed using paired t-test. *P* values: * for *p* ≤ 0.05; ** for *p* ≤ 0.001; *** for *p* ≤ 0.0001.).
