# Supplementary figures and images for: Proteomic analysis of plasma-derived extracellular vesicles: pre- and postprandial comparisons
Source: Sci Rep. 2024 Oct 3;14:23032. doi: 10.1038/s41598-024-74228-4 (PMC11450010; doi:10.1038/s41598-024-74228-4)

Supplementary Figure 1.

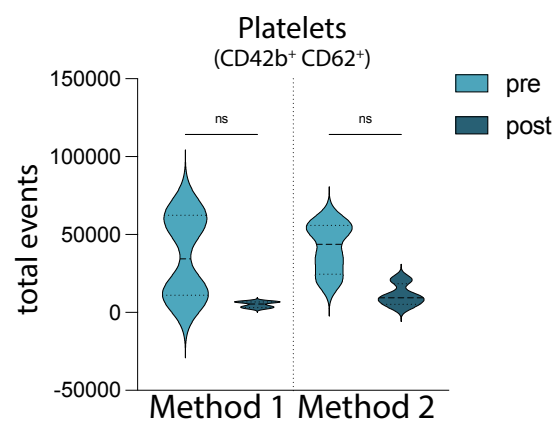

Supplement: Supplementary file 2 — Supplementary Information 2. [file 41598_2024_74228_MOESM2_ESM.pdf]

Supplementary Figure 2.

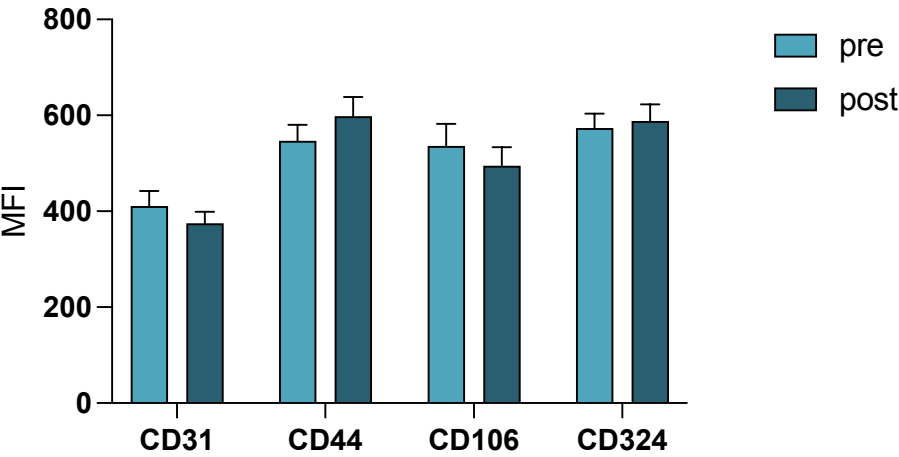

Supplement: Supplementary file 3 — Supplementary Information 3. [file 41598_2024_74228_MOESM3_ESM.pdf]
